# Matrons in Council.—Some Return Sparks: A Matron's View

**Published:** 1917-08-11

**Authors:** 


### THE MATRONS' AND SISTERS' DEPARTMENT

### MATRONS IN COUNCIL.

### SOME RETURN SPARKS: MATRON'S AND SISTER'S VIEWS.

The article entitled "Sparks from a Nurse's Anvil" seems to open a field for some comments drawn from practical experience. The writer has a high idea of matrons' powers and privileges. The notion that a matron, in the "less exposed official region" has a nice easy time and a free hand, is a beautiful illusion, quickly dissipated by experience. The absolute tyrant matron is a thing of the past. Before we reduce hospital discipline any further, perhaps it would be well to consider the example, just shown us in Russia, of an army where saluting was abolished, and where attacks were managed by syndicates instead of commanders. The writer well says that wisdom is not the exclusive possession of officials, though the experience of most of us would make us hesitate to endorse her further remark that it is a universal gift. In any case, we may all, whether official or free-lance, remember with profit the saying of the late Master of Trinity, that "we are none of us infallible, not even the youngest of us."

The future College Council might find it a great advantage to have the Matron-in-Chief of the Army Nursing Service as an *ex-officio* member. Her experience would be very useful, especially in time of war. But any alteration considered to be desirable in Army nursing must come from within. Interference by an external body would probably do more harm than good.

With regard to the present system of nursing education, the examination does not make the nurse, but it is necessary that every trained nurse should have a minimum of theoretical knowledge. When the College of Nursing is fully established, there will probably be an Honours, as well as a Pass, examination. Women whose tastes lie more in the direction of theory than of practice would take honours, and become lecturers in hospital. They would not necessarily be the best matrons: administrative ability is by no means always associated with success in examinations.

The question, raised by the article, of a guarantee of ideals presents greater difficulties. This cannot be secured by examination. It would be quite easy to get up the subject of ideals from books, with no intention of making them everyday realities. Perhaps the nearest approach to a guarantee would be, first, that the College should insist on the production of a certificate from the nurse's training-school as a condition of entrance for the College examination. The matron's marks which are necessary for obtaining the certificate should be made of primary importance. These marks are given for conduct and work during the whole period of training; not only for the last few months, when the nurse is more or less on her guard. It would, happily, be impossible to most girls to *pretend* consistently for three years to have a high standard of work and conduct—to be conscientious, hard-working, and kind and gentle to their patients. They must either acquire these qualities or it must soon appear that they do not mean to do so. In the latter case, they ought not to remain after the first year. At present it is very difficult to insist on this, unless the probationer does something outrageous. If the matron only reports that she is unsatisfactory, careless, and quite uninterested in her patients and her work, *the committee will probably allow her to remain.* Yet the plan of allowing an unsatisfactory nurse to finish her training, and then to fail in her examination and leave without a certificate, is a most unfair one, both to the nurse herself and to the sisters in whose wards she works.

The meaning of the word "education" is to draw out; that is, to develop what already exists in a rudimentary form. Thus we say that a girl has the makings of a good nurse in her. The theory of nursing can be taught by lectures and coaching; but these, without practical experience, will not make a skilled nurse. It is also possible to teach probationers high professional ideals in lectures; but to vitalise and become part of the nurse's life, she must work in the wards under the influence and by the example of good ward sisters. The matron has the responsibility of choosing the sisters, and she should also encourage them to expect help and sympathy from her in their difficulties, and especially in training the probationers. It is important that the matron should understand the probationer's character, its strong points as well as the weak. In large hospitals, at any rate, this can only be done well by means of real confidence between the matron and the sisters. The matron will, of course, check each report of a probationer by her own observation, and by other reports, and will allow for prejudice and for incompatibility of temper. All this is difficult work, and takes a great deal of time. But we are not thinking of a matron who grudges time and trouble taken for her nurses, or who only wishes to do work that will bring her public notice and applause. She is a rare specimen, and one can only recommend her to reform or resign. In almost all training-schools the matron really desires to be a mother to her nurses, and to educate them in the highest ideals of their profession.

